# Targeting EIF4F complex in non-small cell lung cancer cells

**DOI:** 10.18632/oncotarget.18413

**Published:** 2017-06-08

**Authors:** Lu Dai, Zhen Lin, Yueyu Cao, Yihan Chen, Zengguang Xu, Zhiqiang Qin

**Affiliations:** ^1^ Department of Genetics, Louisiana State University Health Sciences Center, Louisiana Cancer Research Center, New Orleans, LA 70112, USA; ^2^ Department of Pediatrics, East Hospital, Tongji University School of Medicine, Shanghai 200120, China; ^3^ Research Center for Translational Medicine and Key Laboratory of Arrhythmias, East Hospital, Tongji University School of Medicine, Shanghai 200120, China; ^4^ Department of Pathology, Tulane University Health Sciences Center, Tulane Cancer Center, New Orleans, LA 70112, USA

**Keywords:** EIF4F, EIF4A, EIF4E, EIF4G, eukaryotic initiation factors

## Abstract

Non-small cell lung cancer (NSCLC) accounts for about 85–90% of lung cancer cases, which represents the leading cause of cancer-related death in the world. The majority of lung cancer patients doesn't respond well to conventional chemo-/radio-therapeutic regimens and have a poor prognosis. The recent introduction of targeted therapy and immunotherapy gives new hopes to NSCLC patients, but their outcome/prognosis is far from satisfactory. The translation initiation EIF4F complex has been shown to play important roles in cancer progression, but its functional role and therapeutic effect in lung cancers especially NSCLC remain largely unknown. In this current review, we summarize recent findings regarding the role of EIF4F complex in NSCLC progression and targeted therapy potentials. We also discuss the unanswered questions and future directions in this field.

## INTRODUCTION

Lung cancer is the leading cause of cancer-related death in the world, which can be classified as small cell lung cancer (SCLC) and non-small cell lung cancer (NSCLC) [1]. NSCLCs account for about 85–90% of lung cancer cases, which encompassing lung adenocarcinomas, lung squamous cell carcinomas, and large cell carcinomas [1]. Clinically, the majority of NSCLC patients doesn't respond well to current chemo-/radio-therapeutic regimens and have a dismal 5-year survival rate of ˜15% [2]. Most recently, introduction of targeted therapy and immunotherapy gives new hopes to NSCLC patients, but the outcome/prognosis is far from satisfactory. For instance, targeted therapeutic drugs such as gefitinib inhibiting mutated epidermal growth factor receptor (EGFR) exhibit good initial effects, but the drug resistance inevitably develops after 10-month of treatment and patients will succumb to this disease [3]. Meanwhile, inhibition of immune checkpoint factors such as PD-1 and PD-L1 has yielded good clinical responses and improved overall survival in certain NSCLC patients, however, only 15–20% of NSCLC patients respond to such therapy and affordability is another serious issue, since a single-course (7-month) treatment will cost more than $100,000 [4, 5]. Thus, there is an urgent need to better understand the mechanisms of lung carcinogenesis and to identify new therapeutic targets for improving the treatment.

The protein synthesis or mRNA translation is tightly controlled in the eukaryotic cells. Dysregulating this process can contribute to the carcinogenesis by affecting key cancer pathways [6–8]. The eukaryotic initiation factor 4F (EIF4F) complex is a heterotrimeric complex composed of a 5′ mRNA cap-binding subunit EIF4E, the large scaffolding protein EIF4G, and the ATP-dependent RNA helicase EIF4A [9]. Meanwhile, the heterogeneity of the EIF4F complex is further increased by the isoforms of individual EIF4 protein (at least 3 isoforms for each EIF4 protein) [9]. The EIF4F complex recruits mRNA to the ribosome then helps the ribosome to scan the 5′ untranslated region (5′ UTR) in search of an initiation codon. Over the last two decades, the EIF4F complex has been shown to play important role in oncogenesis [10, 11]. There are several strategies have been developed for directly targeting EIF4F complex, which include the down-regulation of EIF4E with antisense oligonucleotides (ASOs), disrupting EIF4F complex formation, impeding EIF4E–cap interaction and targeting EIF4A [9]. However, there is limited data about its functions and therapeutic effects in lung cancer especially NSCLC. The current review will summarize recent findings regarding the role of EIF4F complex in NSCLC progression as well as its potential therapeutic value.

### The expression of EIF4F complex components in NSCLC and its prognostic value

In NSCLC, EIF4E is arguably the most studied component of EIF4F complex and serves as a rate-limiting factor of cap-dependent translation initiation. Emerging evidence has shown that EIF4E, especially its phosphorylated form is up-regulated in NSCLC tumor tissues, which is correlated with a shorter survival and increased lymph node metastasis [12–14]. Moreover, EIF4E is also co-regulated with some other tumor-associated proteins in NSCLC. For example, one study shows that EIF4E(+)/cyclin D1(−) NSCLC patients have poorer clinical outcome, while EIF4E(−)/cyclin D1(+) patients have a more favorable outcome, and patients with EIF4E(+)/cyclin D1(+) have an intermediate outcome [15].

Compared to EIF4E, the expression and clinical implication of other components of the EIF4F complex (such as EIF4G and EIF4A) in NSCLC are far less understood. Our group recently reports that the expressional level of EIF4G1 is much higher in NSCLC cell lines and primary tumor tissues, than their normal controls [16]. Meanwhile, another study shows that NSCLC patients with low EIF4A2 expression have worse overall survival and disease-free survival [17]. Thus, varying EIF4F components may play different roles in lung carcinogenesis and more research needs to be engaged to determine the correlation between these EIF4F complex components expression and NSCLC prognosis in the future.

### The EIF4F complex and NSCLC cell survival and proliferation

We recently reports that stably silencing EIF4G1 by shRNA significantly reduces NSCLC cell proliferation and anchorage-independent growth *in vitro*, through inducing apoptosis as well as G_0_/G_1_ cell cycle arrest [16]. Treatment with EIF4E/EIF4G interaction inhibitor, 4EGI-1, can dramatically inhibit the cell growth and induce apoptosis in NSCLC cell cultures [18]. Moreover, 4EGI-1 can enhance the apoptotic effects of tumor necrosis factor-related apoptosis-inducing ligand (TRAIL) on NSCLC cells, by inducing CCAAT/enhancer-binding protein homologous protein-dependent DR5 and ubiquitin/proteasome-mediated degradation of cellular FLICE-inhibitory protein (c-FLIP) [18]. Meanwhile, EIF4E can be directly targeted by the tumor suppressor miR-34c-3p and knock-down of EIF4E inhibits NSCLC cell proliferation [19]. In accord with this, miR-34c-3p expression is significantly decreased in NSCLC patient biopsies as well as cancer cell lines, which thus allows overexpressed EIF4E to promote lung carcinogenesis [19]. Along this line, a recent study shows that antisense oligonucleotide targeting EIF4E (4EASO) significantly inhibits NSCLC cell proliferation as well as the expression of several oncogenic proteins such as VEGF, c-MYC and Osteopontin [20].

### The EIF4F complex and NSCLC metastasis

Metastasis reflects the invasiveness of tumor cells and serves as a key prognosis factor for NSCLC. Our recent data show that stably silencing EIF4G1 reduces NSCLC cell invasion and migration [16], indicating an important role of EIF4 factors in NSCLC metastasis. Consistently, Li *et al*. have reported that elevated expression of EIF4E in NSCLC is associated with a stronger tumor invasion [21]. Another study confirms that knock-down of EIF4E or EIF4G1 prevents NSCLC cell migration and epithelial-to-mesenchymal transition (EMT), which represent essential steps for tumor metastasis [22]. Interestingly, secretome of human bone marrow mesenchymal stem cells can simultaneously decrease the levels of EIF4E and EIF4G1 and attenuate the invasiveness of NSCLC cells [23], which further supports the notion that EIF4E/G1 likely play important roles in NSCLC metastasis. In accord with this, miR-34c-3p mediated knock-down of EIF4E also inhibits NSCLC cell migration and invasion [19].

### The EIF4F complex and NSCLC chemoresistance

The acquisition of multidrug resistance is a major reason to cause a failure of chemotherapy in NSCLC patients. Accumulating evidence has shown that the EIF4F complex components are involved in NSCLC multidrug resistance. For example, elevated expression of EIF4E is tightly linked to NSCLC resistance to erlotinib, gefitinib, cisplatin, and gemcitabine [20, 21, 24, 25]. Interestingly, several EIF4-targeting miRNAs are likely to be involved in the chemoresistance in NSCLC. Hao *et al*. recently reported that the suppression of EIF4G2 by miR-379 sensitizes NSCLC cells to cisplatin [26]. However, Wang *et al*. reported that suppression of EIF4E by miR-141 increases NSCLC cell resistance to docetaxel and the expression of EIF4E in docetaxel chemoresistant NSCLC patients is markedly lower than those of docetaxel sensitive NSCLC patients [27]. Therefore, it is unclear whether the individual EIF4F complex components may play different roles in the resistance of varying therapeutic agents in NSCLC.

### The crosstalk between the EIF4F complex and intracellular signaling and partners in NSCLC

The formation and function of the EIF4F complex need to be tightly regulated to maintain the cellular homeostasis. Some major signal transduction pathways (e.g., PI3K/Akt/mTOR or MAPK) [28–30] and transcription factors (e.g., MYC or C/EBPα) have been shown to regulate the EIF4F complex [31, 32]. In NSCLC, the rapamycin mediated inhibition of mTOR signaling can increase the phosphorylation of both Akt and EIF4E through a negative feedback mechanism, while the PI3K inhibitors such as LY294002 can reverse such undesirable effects [33]. Other studies also demonstrate that simultaneous inhibition of PI3K/Akt and mTOR signaling exerts synergistic anticancer activity against NSCLC *in vitro* and *in vivo* [34, 35]. Another study reveals that the c-jun N-terminal kinase (JNK) can activate cap-dependent translation in NSCLC cells as an alternative pathway, which is independent of PI3K/Akt/mTOR signaling [36].

Using tandem affinity purification combined with mass spectrometry (TAP-MS) screening approach, we have identified that ubiquitin-specific protease 10 (USP10) can directly interact with EIF4G1 and inhibit EIF4G1 activities in NSCLC cells [16], although the underlying mechanism remains largely unknown and further investigations are warrant to solve this puzzle.

## CONCLUSIONS

**Figure 1:**
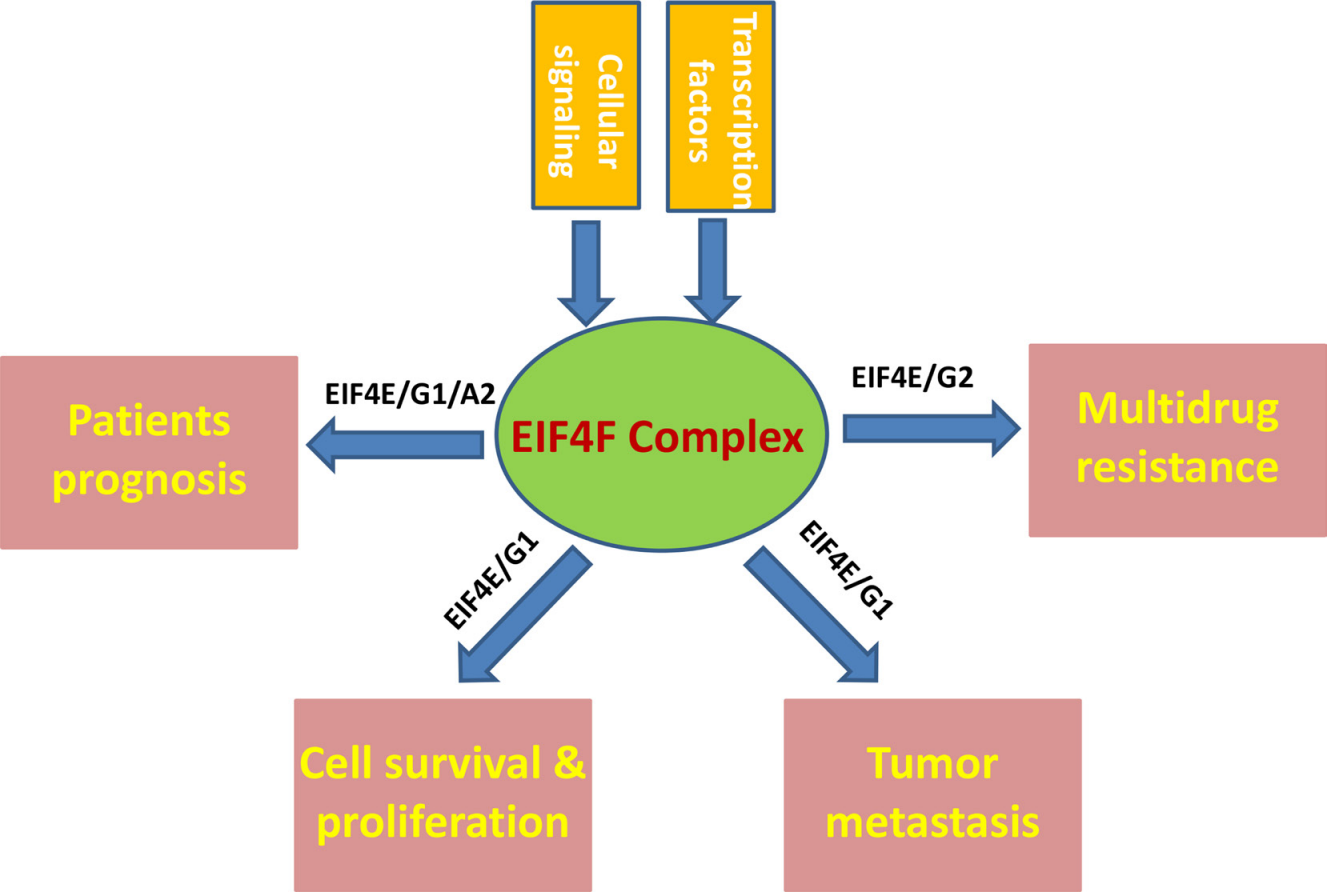
Schematic of known and putative contributions of EIF4F complex to NSCLC pathogenesis

Even though the recent targeted therapy and immunotherapy improve the average survival of a small group of lung cancer patients, the initial non-response, acquired resistance, recurrence are still serious problems faced by the majority of NSCLC patients. Furthermore, given the high heterogeneity of lung cancer, the monotherapy usually does not work for most patients. As the components of the translation machinery integrate almost all oncogenic signals [9], targeting the EIF4F complex components holds the promise for overcoming a major hurdle associated with intra-tumor heterogeneity. Moreover, because malignant cells exhibit augmented activity of most of the EIF4F complex components, it is hypothesized that the tumor cells become ‘addicted’ to elevated protein synthesis and more sensitive to translation machinery targeted therapy [37, 38]. Unlike other solid tumors, there are much fewer data about the expression, regulation, and biological roles of the EIF4F complex components in NSCLC, which have been summarized in Figure 1. Several key questions remain unanswered and need to be addressed in the future: *1)* there is still lacking of large-scale clinical studies to investigate the expression of EIF4F complex components especially different isoforms in NSCLC biopsies, as well as their correlation with tumor stages, histology, metastasis and prognosis. *2)* are the expression and/or functions of EIF4F complex components affected by genetic landscape of the NSCLCs such as mutation of the EGFR, HER2, and MET genes as well as fusion oncogenes involving anaplastic lymphoma kinase (ALK), ROS1 and RET [39, 40]? *3)* A more detailed regulatory/interaction network for EIF4F complex in NSCLC is still missing. *4)* more effective EIF4F complex inhibitors or other targeted therapeutic strategies need to be developed to accelerate the “bench to bedside” switch.
